# ClinicRealm: Re-evaluating large language models with conventional machine learning for non-generative clinical prediction tasks

**DOI:** 10.1038/s41746-026-02539-z

**Published:** 2026-04-08

**Authors:** Yinghao Zhu, Junyi Gao, Zixiang Wang, Weibin Liao, Xiaochen Zheng, Lifang Liang, Miguel O. Bernabeu, Yasha Wang, Lequan Yu, Chengwei Pan, Ewen M. Harrison, Liantao Ma

**Affiliations:** 1 School of Artificial Intelligence, Beihang University, Beijing, China; 2 National Engineering Research Center for Software Engineering, Peking University, Beijing, China; 3 School of Computing and Data Science, The University of Hong Kong, Hong Kong SAR, China; 4 Centre for Medical Informatics, The University of Edinburgh, Edinburgh, UK; 5 Health Data Research UK, London, UK; 6 School of Computer Science, Peking University, Beijing, China; 7 ETH Zurich, Zurich, Switzerland

**Keywords:** Outcomes research, Prognosis

## Abstract

Large Language Models (LLMs) are increasingly deployed in medicine. However, their utility for non-generative clinical prediction is under-evaluated, and they are often assumed to be inferior to specialized models, creating potential for misuse and misunderstanding. To address this, our ClinicRealm benchmark systematically evaluates 15 GPT-style LLMs, 5 BERT-style models, and 11 traditional methods on unstructured clinical notes and structured Electronic Health Records (EHR) across predictive performance, reasoning, fairness, etc. Our findings reveal a significant shift: on clinical notes, leading zero-shot LLMs (e.g., DeepSeek-V3.1-Think, GPT-5) now decisively outperform finetuned BERT models. On structured EHRs, while specialized models excel with ample data, advanced LLMs demonstrate potent zero-shot capabilities, often surpassing conventional models in data-scarce settings. Notably, leading open-source LLMs match or exceed their proprietary counterparts. This provides compelling evidence that modern LLMs are competitive tools for clinical prediction, necessitating a re-evaluation of model selection strategies by health data scientists and developers.

## Introduction

In modern healthcare, the ability to accurately predict and understand patient outcomes using vast amounts of healthcare data is critical. A spectrum of data, from unstructured clinical notes to structured Electronic Health Records (EHR), presents a complex landscape for analysis. Clinicians increasingly utilize machine learning (ML) and deep learning (DL) models for informed decision-making, leveraging models trained specifically for tasks like mortality and hospital readmission prediction^1–3^. More recently, the advent of generative artificial intelligence (GenAI), particularly Generative Pre-Trained Transformer-based Large Language Models (GPT-style LLMs), has shown remarkable capabilities in various generative tasks, including patient interaction^4^, resolving complex medical queries^5^, and passing medical licensing exams^6^, sparking interest in their broader clinical potential^7^.

While LLMs have excelled in generative tasks, their effectiveness in non-generative clinical tasks, such as risk probability assessment and outcome classification, crucial for direct clinical decision support, has been less clear and often underestimated. Indeed, a prevailing assumption has been that LLMs generally underperform these specialized, locally trained models in such clinical prediction scenarios^8–10^. However, our comprehensive benchmarking of state-of-the-art LLMs, including reasoning-enhanced and open-source models, challenges this view and reveals a rapidly evolving landscape. Our findings highlight several key aspects of this evolution:

- For unstructured clinical notes, where locally finetuned BERT variants were widely considered optimal for prediction^9–11^, our results indicate a significant shift. Recent zero-shot LLMs (e.g., GPT-5, DeepSeek-R1/V3.1) now substantially outperform these specialized models, suggesting that the extensive efforts for BERT finetuning in this domain may be less critical than previously thought.
- Regarding structured EHR data, conventional ML/DL models perform strongly when trained with ample data^3,8^. In contrast, advanced LLMs (e.g., GPT-5, DeepSeek-R1/V3.1) show impressive zero-shot capabilities; even when compared to simpler models trained on full datasets, their performance can be within a 10% margin. In data-scarce scenarios, these LLMs can surpass most conventional models. This makes them particularly promising for applications with limited data, such as in the context of emerging diseases or rare conditions.
- Furthermore, the performance of medically domain-specific finetuned LLMs in these predictive tasks warrants careful consideration. While often optimized and finetuned for question-answering, our experiments show they may not offer an advantage over general-purpose LLMs for non-generative, discriminative clinical predictions. Notably, leading open-source models (e.g., DeepSeek) demonstrate performance in these tasks that is comparable, and at times superior, to proprietary counterparts, further broadening the accessibility of high-performing models in data-sensitive settings.

**Fig. 1:**
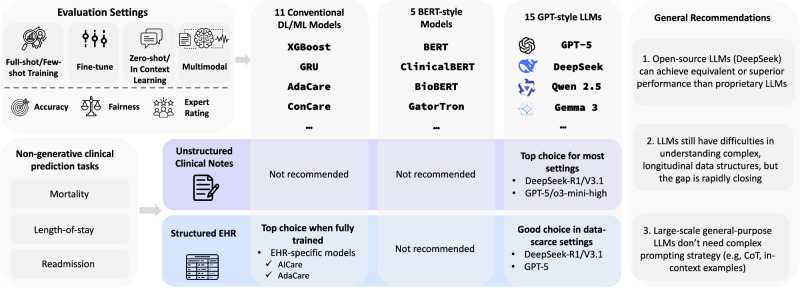
Comparative performance and recommendations for model selection in non-generative clinical tasks. This figure summarizes the benchmarking results, offering guidance on selecting optimal models for different clinical scenarios. It compares conventional DL/ML, BERT-style, and Large Language Models (LLMs) across two categories of tasks: (i) prediction tasks using unstructured clinical notes data (e.g., mortality, readmission prediction), and (ii) prediction tasks using structured Electronic Health Record (EHR) data (e.g., mortality, length-of-stay prediction).

The evidence for these observations is drawn from our ClinicRealm benchmark, which evaluates 15 state-of-the-art Large Language Models (LLMs), including the latest high-performing open-source and proprietary releases, alongside 5 BERT-style language models representing previously leading text encoders. These are further benchmarked against 11 established conventional machine learning/deep learning models, including 4 specialized predictive models designed for EHR data, ensuring a comprehensive assessment across the spectrum of current and foundational methodologies. Furthermore, we extend our analysis to multimodal settings, evaluating models that integrate both structured EHR data and unstructured clinical notes. This study aims to rectify existing potential misconceptions and provide clarity across representative non-generative medical prediction tasks (Fig. 1). By offering actionable insights into optimal model selection for various clinical data types and tasks, from structured EHR to clinical notes, we identify key research gaps for future clinical LLM development. Ultimately, this work seeks to enhance the quality of patient care by facilitating more accurate and appropriately chosen predictive analytics. We provide the up-to-date benchmark results online (https://yhzhu99.github.io/ehr-llm-benchmark/).

## Results

Our subsequent analysis provides a comprehensive benchmark of LLMs in clinical settings. We structure our investigation to first evaluate predictive performance across fundamental data modalities: unstructured clinical notes, where LLMs’ linguistic capabilities are tested, and structured EHR data, a critical but less-explored domain for these models. We then assess their ability to integrate these sources in multimodal prediction tasks. Moving beyond quantitative accuracy, our evaluation delves into the qualitative aspects of model behavior through a human-led expert review of their clinical reasoning and a systematic analysis of common error patterns.

### Benchmarking results of prediction tasks using clinical notes data

Our analysis of prediction tasks using unstructured clinical notes distinguishes between clinically relevant prospective prediction and retrospective document classification, revealing the robust capabilities of modern LLMs in both settings (Table 1).

**Table 1:** Performance comparison of BERT-style and GPT-style models on MIMIC-III mortality, MIMIC-IV mortality and readmission prediction tasks using unstructured clinical notes

	

**Bold** denotes the best performance among all baselines of all settings. We use a bootstrapping strategy on all test set samples 100 times to report the mean ± std results. The values in brackets represent the 95% confidence interval, with the lower and upper bounds corresponding to the 2.5 and 97.5 percentiles, respectively. All metrics are multiplied by 100 for readability purposes.

For the methodologically rigorous prospective in-hospital mortality task, which uses only admission notes from the first 24 h (MIMIC-III), state-of-the-art LLMs demonstrated powerful zero-shot predictive capabilities. As anticipated, this task proved more challenging than retrospective analysis, resulting in generally lower performance. We find that leading LLMs outperformed specialized, finetuned BERT-style models. The DeepSeek-R1 achieved the highest AUROC (90.75%), representing a significant improvement over the 87.97% AUROC from the finetuned GatorTron model, a strong clinical NLP baseline. Other advanced models, including the newly released GPT-5 (89.75% AUROC), and DeepSeek-V3.1-Think (88.42% AUROC), also delivered highly competitive performance, surpassing the finetuned specialist model. Notably, this superiority holds even though the specialized BERT baselines (e.g., GatorTron, ClinicalBERT) included MIMIC-III notes in their pretraining data, giving them a theoretical advantage of domain exposure, whereas the evaluated LLMs were applied zero-shot. These results provide compelling evidence that the predictive power of modern LLMs extends to challenging, clinically realistic prospective scenarios.

In the retrospective document classification tasks using MIMIC-IV discharge summaries, which assess a model’s ability to synthesize information from a complete hospital record (serving as a proxy for automated coding), the latest generation of LLMs achieved exceptionally high performance in a zero-shot setting. For mortality classification, DeepSeek-V3.1 attained a near-perfect AUROC of 97.89%, followed closely by GPT-5 (97.6%) and o3-mini-high (97.45%). For 30-day readmission prediction, a prospective task predicted at discharge, o3-mini-high (87.59% AUROC) and GPT-5 (86.34% AUROC) demonstrated top performance. In all note-based tasks, these zero-shot LLM results substantially surpassed the best finetuned BERT-style model, GatorTron.

Across both prospective and retrospective evaluations, a key observation is the consistent high performance of open-source models. While proprietary models like GPT-4o and GPT-5 performed strongly, open-source competitors such as DeepSeek-R1 and DeepSeek-V3.1 achieved comparable or superior results. This is particularly relevant for healthcare, as it enables organizations to leverage cutting-edge AI for clinical prediction while maintaining full control over sensitive patient data through secure on-premise deployments.

Regarding model settings, for the most advanced LLMs, the zero-shot prompt setting consistently yielded the best results, underscoring their powerful out-of-the-box clinical text understanding. For less advanced LLMs (e.g., Qwen2.5-7B, HuatuoGPT-o1-7B), finetuning generally improved performance over using pretrained embeddings (freeze setting) or direct prompting. As noted previously, several older or smaller finetuned LLMs (e.g., BioGPT, Meditron, GPT-2) failed to follow prompts correctly and were excluded from the “prompt” evaluation.

### Benchmarking results of prediction tasks using structured EHR data

Our benchmarking reveals that state-of-the-art LLMs, notably GPT-5 and DeepSeek-V3.1-Think, are capable of performing zero-shot clinical predictions directly from structured EHR data (Table 2). However, their performance in this zero-shot setting generally remains below that of conventional machine learning (ML) and deep learning (DL) models trained on the complete dataset (full shot). Specialized DL models like AdaCare and AICare, when fully trained, consistently achieved the highest performance across most tasks on both datasets.

**Table 2:** Performance of mortality and readmission prediction on TJH and MIMIC-IV datasets using structured EHR

	

**Bold** indicates the best performance excluding results using the full dataset. *Italic* indicates the proposed prompting framework outperforms basic prompts. We use bootstrapping on all test set samples 100 times to report the mean ± std results. The values in brackets represent the 95% confidence interval, with the lower and upper bounds corresponding to the 2.5 and 97.5 percentiles, respectively. All metrics are multiplied by 100 for readability purposes.

A key finding emerges when comparing zero-shot LLMs to traditional models trained with limited data (10 shot). On the more complex MIMIC-IV dataset, top-performing LLMs demonstrated remarkable strength. Specifically, GPT-4o using an optimized prompt with in-context learning (opt.+ICL) achieved a zero-shot AUROC of 85.99% for mortality prediction, surpassing all evaluated 10-shot conventional models, including the best performing 10-shot model, AdaCare (80.02% AUROC). This highlights the potent data-efficiency of leading LLMs for certain clinical prediction tasks. Conversely, on the TJH dataset tasks, even 10-shot conventional models (e.g., CatBoost achieving 99.43% AUROC for outcome prediction) reached near-perfect performance, there was less room for zero-shot LLMs to demonstrate an advantage. This performance disparity is likely attributable to inherent differences in task complexity and data heterogeneity, a point analyzed further in Supplementary Information.

We evaluated LLMs using three prompt strategies: a basic sequential feature input (base prompt), an optimized prompt with better formatting and instructions, and an optimized prompt with in-context learning (opt.+ICL). The impact of these strategies was highly variable, depending on the model, task, and dataset complexity. For instance, on the challenging MIMIC-IV mortality task, adding in-context learning significantly boosted GPT-4o’s performance from 76.18% AUROC (optimized) to 85.99% (opt.+ICL). In contrast, for some large-scale models, increased prompt complexity occasionally led to diminished performance; GPT-5’s AUROC on the TJH outcome task, for example, decreased when moving from a base to an optimized prompt. While not always improving accuracy metrics, prompt engineering was critical for model reliability. As detailed in our failure analysis in Supplementary Information, using an optimized prompt often dramatically reduced the “failure-to-predict” rate for models like HuatuoGPT-o1-7B and Gemma-3-4B, ensuring they produced a valid output.

Among the LLMs tested, GPT-5 and the large-scale DeepSeek-V3.1-Think consistently demonstrated superior zero-shot classification capabilities compared to smaller models like Qwen2.5-7B, Gemma-3-4B, and specialized models like HuatuoGPT-o1-7B. On the MIMIC-IV dataset, the open-source DeepSeek-V3.1-Think achieved an AUROC equivalent to or even higher than that of GPT-5. Similar to our note-based experiments, we encountered challenges with prompt adherence for several models, particularly those finetuned on specialized corpora, which often deviated from the requested output format and were subsequently removed from the table.

While a performance gap persists between zero-shot LLMs and fully trained conventional models, the strong few-shot and zero-shot performance of models like DeepSeek underscores their potential value in data-scarce clinical scenarios, such as predicting outcomes for emerging diseases or in settings with limited historical data.

### Benchmarking results of multimodal prediction tasks

Our multimodal experiments, which integrate both structured EHR data and unstructured clinical notes, yield a complex and nuanced picture of performance, as detailed in Table 3. The results reveal that combining data modalities does not uniformly lead to improvements, with outcomes dependent on the integration method, the clinical task, and the inherent predictive power of each data source. For finetuned models, we use the best-performing unimodal encoders identified in our main experiments:

- For the **in-hospital mortality task**, based on their strong unimodal performance, we selected AdaCare as the encoder for structured EHR data and a finetuned GatorTron for clinical notes.
- For the **30-day readmission task**, we selected LSTM for structured EHR data and a finetuned HuatuoGPT-o1-7B for clinical notes.

**Table 3:** Performance of mortality prediction and MIMIC-IV datasets using multimodal EHR data

	

**Bold** indicates the best performance excluding results using the full dataset. *Italic* indicates the proposed prompting framework outperforms basic prompts. We use bootstrapping on all test set samples 100 times to report the mean ± std results. The values in brackets represent the 95% confidence interval, with the lower and upper bounds corresponding to the 2.5 and 97.5 percentiles, respectively. All metrics are multiplied by 100 for readability purposes.

Feature-fusion models show competent but not superior performance. Our feature-fusion approaches demonstrate that structured integration can yield strong predictive models. For in-hospital mortality, the Self-Attention fusion model achieved an AUROC of 95.62%, a slight improvement over the best-performing unimodal structured EHR model (AdaCare, 94.28%). However, this result does not surpass the performance of the top-performing LLM on clinical notes alone (DeepSeek-V3.1, 97.89%). A similar trend was observed for 30-day readmission, where the best fusion model (Cross-Attention, 82.18%) performed comparably to the best unimodal structured EHR model (LSTM, 82.52%) but failed to match the best note-based model (o3-mini-high, 87.59%). This suggests that for these tasks, unstructured clinical notes are an exceptionally rich source of information, and while feature fusion effectively leverages both data types, it struggles to add significant value when one modality is already dominant.

Prompt-based multimodal integration yields complex outcomes for LLMs. A key finding is that providing LLMs with combined structured and unstructured data in a single prompt does not straightforwardly improve upon their best unimodal performance. The multimodal performance is generally positioned between the LLMs’ performance on structured EHR data alone and their performance on clinical notes alone. For instance, in mortality prediction, GPT-5’s multimodal AUROC of 92.03% improves upon its EHR-only performance (81.25%) but remains substantially below the level achieved using clinical notes alone (97.60%). Similarly, for readmission, its multimodal AUROC of 73.75% is better than its EHR-only performance (65.65%) but represents a considerable drop from its note-only performance (86.34%). This indicates that while the models can process the combined input, they fail to effectively synthesize the information to reach the high predictive accuracy offered by the clinical narrative.

The observed performance gap suggests that current LLMs face challenges in zero-shot multimodal synthesis, such as attentional bias or distribution shifts. To investigate the mechanisms behind this underperformance, we conducted additional ablation studies on data formatting (converting structured lists to natural language text) and modality ordering (see Supplementary Information), revealing that performance is highly sensitive to the interaction between information layout and data distribution. For example, we found that the standard list-style EHR format functions best when placed before the note (EHR-first), likely acting as a “system context”, whereas converting structured data into natural language sentences (text-style) is essential for maintaining performance when notes are presented first. However, even with these optimizations, multimodal integration often failed to surpass the strong unimodal baseline of clinical notes.

### Human evaluation of LLM reasoning quality

Our human evaluation by five clinical experts revealed that the reasoning provided by top-performing LLMs was generally of high quality and clinically relevant. The quantitative scores, summarized in Table 4, show that the reasoning quality of the top-performing LLMs is generally decent. We quantified agreement among the five clinicians using Fleiss’ Kappa. Across tasks/dimensions, the Fleiss’ Kappa values range from 0.26 to 0.79, indicating fair to substantial agreement overall, with particularly strong agreement in several settings (e.g., structured EHR mortality for Clinical Accuracy & Safety, *κ* = 0.79).

**Table 4:** Human evaluation of LLM reasoning quality across clinical prediction tasks

Task (MIMIC-IV)	Clinical Accuracy & Safety	Reasoning & Completeness	Clarity & Clinical Utility
*Structured EHR Data*			
In-hospital Mortality	3.41 ± 1.00 (0.79)	4.14 ± 0.67 (0.26)	4.03 ± 0.76 (0.57)
30-day Readmission	3.60 ± 1.14 (0.41)	4.30 ± 0.73 (0.67)	4.05 ± 1.19 (0.35)
*Clinical Notes Data (Discharge Summary)*			
In-hospital Mortality	4.30 ± 1.13 (0.59)	4.60 ± 0.68 (0.37)	4.65 ± 0.59 (0.43)
30-day Readmission	2.80 ± 1.57 (0.72)	3.93 ± 1.22 (0.55)	3.13 ± 1.46 (0.66)

Mean scores ± standard deviation are reported on a 1–5 scale, where 5 represents the highest quality. Values in parentheses represent the Fleiss’ Kappa for inter-rater agreement. The best-performing LLM from our main experiments was evaluated for each task.

For unstructured clinical notes for mortality prediction, the reasoning was rated as highly accurate, complete, and useful (mean scores of 4.30, 4.60, and 4.65, respectively). For structured EHR data, the reasoning was also rated favorably, with “Reasoning & Completeness” scoring above 4.0 for both tasks. This indicates the models are effective at identifying key risk factors from tabular data and logically connecting them to the prediction. However, the “Clinical Accuracy & Safety” scores were moderately lower (3.41 and 3.60), reflecting instances where models occasionally misinterpreted or hallucinated minor details from the dense numerical input.

Notably, the most challenging setting for the LLM was predicting 30-day readmission from clinical notes. This task received the lowest scores across all dimensions, particularly for accuracy (2.80) and utility (3.13). This suggests that predicting readmission from a discharge summary requires a more nuanced synthesis of clinical and social factors, a task where the models’ reasoning is currently less reliable and more prone to errors.

**Fig. 2:**
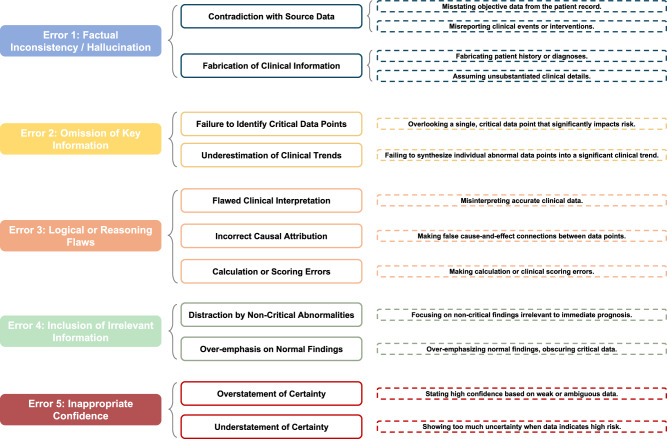
Hierarchically structured error taxonomy for LLM-generated clinical reasoning. This taxonomy was developed by two expert clinicians using an open-coding thematic analysis of a pilot set of model outputs. It provided a standardized framework for the detailed error analysis.

To move beyond aggregate performance metrics and understand the specific failure modes of LLMs in clinical prediction, we conducted a systematic error analysis of their generated reasoning. Leveraging our human evaluation framework and a clinician-developed error taxonomy (Fig. 2), we analyzed the common patterns of the confusion matrix. Our findings, summarized in Table 5, reveal distinct and clinically significant cognitive errors. Our key findings are as follows:

- **False Positives (FPs) are primarily driven by factual inconsistency**. The most frequent error in FP cases was “Factual Inconsistency/Hallucination”. In these instances, the model often justified a high-risk prediction by citing incorrect data or hallucinating a non-existent comorbidity, effectively creating a high-risk narrative based on flawed evidence.
- **False Negatives (FNs) are characterized by flawed clinical reasoning**. As confirmed by our deep-dive, the defining error in FN cases was “Flawed Logic or Reasoning”. The models consistently identified the correct risk factors but failed to appreciate their collective weight, leading to a dangerous underestimation of the patient’s true risk. This suggests a weakness in higher-order clinical judgment rather than data extraction.
- **Errors persist in correct predictions** Critically, errors were also present, albeit at lower frequencies, in correctly classified cases (TPs and TNs). For example, a model might correctly predict a high-risk outcome (TP) but base its reasoning on a minor factual inconsistency. This underscores the importance of evaluating the reasoning process, as a model that is correct for the wrong reasons is unreliable and poses risks in a clinical setting.

**Table 5:** Frequency of error types in LLM reasoning across different prediction outcomes

Error Type	True Positives (TP)	True Negatives (TN)	False Positives (FP)	False Negatives (FN)
Factual Inconsistency	0.0%	0.0%	28.3%	0.0%
Omission of Key Information	0.0%	0.0%	3.3%	0.0%
Flawed Logic or Reasoning	0.0%	4.2%	0.0%	100.0%
Inclusion of Irrelevant Info.	0.0%	0.0%	0.0%	0.0%
Inappropriate Confidence	6.7%	4.2%	13.3%	100.0%

Values represent the percentage of cases within each outcome category where the specified error was identified by at least one expert evaluator. The denominator for each column is the total number of cases sampled for that category (TP: [*n* = 15], TN: [*n* = 24], FP: [*n* = 60], FN: [*n* = 1]).

In summary, this detailed error analysis provides a deeper understanding of LLM predictive behavior, showing that FPs and FNs often stem from fundamentally different types of cognitive failures: flawed synthesis versus incomplete information gathering. These insights are crucial for guiding future research toward improving model reliability and are a vital step toward the safe and effective deployment of LLMs in clinical decision support.

## Discussion

Our comprehensive benchmarking reveals a significant shift in the capabilities of LLMs for non-generative clinical prediction tasks, challenging the prevailing assumption of their general inferiority to specialized models. These findings necessitate a re-evaluation of the role of BERT-style models in clinical prediction. While previously considered strong contenders for clinical NLP, our results show they are now significantly outperformed by newer generation LLMs for prediction tasks based on clinical notes. Although finetuned BERT models might still offer a practical alternative where computational resources are extremely constrained or the deployment of very large LLMs is infeasible, their performance advantage has diminished in the face of rapidly advancing LLM capabilities. Conventional machine learning and EHR-specific deep learning models, however, maintain their top-tier status for structured EHR data predictions when sufficient training data is available, excelling at understanding numerical values and recognizing complex temporal patterns inherent in such datasets.

Beyond predictive performance, our study underscores the importance of evaluating the reasoning behind LLM predictions, a critical factor for clinical adoption. Our human evaluation demonstrated that the explanations from top-performing LLMs were rated highly by clinical experts for their accuracy, completeness, and utility. This validates our methodological choice to prompt for reasoning and suggests that these models can produce outputs that are not just numerically correct but also clinically coherent and useful for decision support. However, this evaluation also served to identify specific, recurring error patterns. While the overall quality was high, instances of factual inconsistency, omission of key risk factors, and flawed logic were still present. This highlights the reality that while modern LLMs are remarkably capable, they are not infallible. A detailed analysis of these failure modes is crucial for understanding their limitations and guiding future research toward improving the reliability and trustworthiness of LLM reasoning in high-stakes medical applications. We also observed important behavioral characteristics of LLMs related to practical deployment. Our analysis of failure-to-predict rates revealed significant variability in model reliability. Consistent with findings in the broader LLM literature, prompt adherence remains a challenge, particularly for smaller or less advanced models. While state-of-the-art models like GPT-5 and DeepSeek-V3.1 demonstrated near-perfect reliability, others struggled, especially with complex prompts or unstructured data. We found that effective prompt engineering was critical for improving reliability, though overly complex prompts could sometimes be counterproductive. We also identified unique failure modes, such as excessive verbosity leading to truncated outputs. These findings underscore that practical deployment requires evaluating not just accuracy but also robustness and instruction-following capability. This reinforces the need for LLM research to expand beyond medical Q&A to include finetuning and evaluation on diverse data types and emphasizes that extensive empirical testing and validation are crucial before deployment in critical clinical applications.

Critically, our results regarding the underperformance of prompt-based multimodal integration challenge the prevailing hypothesis that simply aggregating all available data modalities into a massive context window automatically yields superior predictive performance. This finding serves as a cautionary tale against the “more is better” fallacy in clinical context construction. Our analysis suggests that naive multimodal concatenation can introduce noise that dilutes the strongest predictive signals available in the data, rather than reinforcing them. Consequently, effective multimodal context building remains a human-in-the-loop process, where clinical expertise is essential to determine (i) which signals from each modality are actually relevant for a specific endpoint, and (ii) how they should be summarized and presented so that models do not overweight weak or misleading cues. If true multimodal gains are desired, learned fusion (or other task-specific integration mechanisms) may be more reliable than naive concatenation in zero-shot settings.

Furthermore, the responsible deployment of predictive models in healthcare necessitates a rigorous evaluation of their ethical implications, particularly regarding fairness and potential biases across patient subgroups. To address this, we conducted a comprehensive fairness analysis across demographic attributes (Age, Gender, Race), with the full methodology and results detailed in Supplementary Information. Our analysis revealed several critical trends. First, state-of-the-art LLMs in zero-shot settings generally demonstrated greater fairness, with metrics for disparate impact and equal opportunity closer to ideal values compared to many conventional ML/DL models. Second, we observed that conventional models, while achieving high accuracy when trained on the full dataset, sometimes amplified underlying biases present in the data, particularly concerning age and gender. Third, the process of finetuning language models, though often beneficial for task-specific accuracy, was found to occasionally introduce or exacerbate fairness disparities, likely by overfitting to spurious demographic correlations in the training data. Finally, our results suggest that prompt engineering can serve as a tool for bias mitigation; the use of optimized prompts and in-context learning often not only improved predictive performance but also led to more equitable outcomes. These findings collectively underscore that predictive accuracy is an incomplete measure of a model’s clinical utility. A holistic assessment should include a thorough fairness audit. While no model was perfectly equitable, the promising fairness characteristics of thoughtfully prompted LLMs, coupled with the risks associated with conventional training and finetuning, highlight the need for ongoing research into developing and validating models that are not only accurate but also fair and trustworthy.

Despite these promising results, our study has limitations that point toward important future directions. The first of which is around the limited task and dataset diversity. Our structured data evaluations were confined to mortality and readmission predictions using text from the MIMIC-III, MIMIC-IV, and TJH datasets. Investigating the integration of additional data modalities (e.g., medical imaging and genomics) and diverse clinical tasks (e.g., diagnosis prediction, treatment recommendation, and adverse event forecasting) could provide a more comprehensive assessment of model efficacy. The second limitation is around model selection. Although our study included a comprehensive range of models, the rapid advancements in the field may lead to the development of new more effective models after our study period. We do believe however, that these results capture a generalizable conclusion reflecting the underlying architecture of these approaches. However, continuous incorporation of emerging state-of-the-art models will ensure that benchmarks remain relevant and informative. These limitations uncover several critical research gaps for the broader clinical LLM research. There is a pressing need to broaden training and evaluation for robust non-generative capabilities, as most clinical LLM development is still heavily skewed towards medical literature finetuning and generative task evaluation. Future research could also focus on improving LLM comprehension of complex longitudinal EHR data, where they still lag behind specialized models when ample data is available; this could involve novel architectures, specialized pretraining, or hybrid approaches. Finally, addressing deployment complexity, efficiency, and trustworthiness through research into model compression, efficient inference, and privacy-preserving methods is essential for responsible integration into clinical practice.

In conclusion, our ClinicRealm benchmark provides preliminary evidence that modern LLMs have emerged as competitive tools for non-generative clinical prediction. Contrary to previous assumptions, recent LLMs demonstrate state-of-the-art performance on prediction tasks using clinical notes and show considerable promise for structured EHR data, especially in data-limited situations or for rapid initial model deployment. Healthcare providers and institutions should recognize these advancements. While specialized conventional models remain the gold standard for structured EHR predictions given sufficient data, the narrowing performance gap and the unique strengths of LLMs (e.g., zero-shot learning from text, handling unstructured data) position them as powerful tools in the clinical analytics arsenal. The strong performance of open-source LLMs further democratizes access to these capabilities. Policymakers and researchers should foster an environment that supports the continued development, rigorous evaluation, and responsible integration of LLMs, focusing on enhancing their reliability, efficiency, and understanding of complex medical data. Rather than a blanket preference for one model type, optimal clinical decision support will likely involve a nuanced selection strategy, leveraging the distinct strengths of conventional models, BERT-style approaches (where appropriate for resource constraints), and the burgeoning capabilities of modern LLMs.

## Methods

### Data sources and processing

Our study utilizes three publicly available datasets: MIMIC-IV^12^, MIMIC-III^13^, and a dataset from Tongji Hospital (TJH)^14^. All research was performed in accordance with the Declaration of Helsinki. The study utilized publicly available, de-identified datasets. The use of the MIMIC-III and MIMIC-IV datasets was approved by the Institutional Review Boards of the Massachusetts Institute of Technology (Cambridge, MA) and Beth Israel Deaconess Medical Center (Boston, MA), and the requirement for individual patient consent was waived for these de-identified datasets. The TJH dataset is also a public, de-identified dataset for which consent was waived. This study was conducted in compliance with the respective data use agreements for all datasets.

- The **TJH dataset**^14^ is derived from Tongji Hospital and is publicly available on GitHub. It comprises structured EHR data for 485 COVID-19 patients admitted during the initial COVID-19 outbreak, including 73 numerical lab tests and vital signs, plus two demographic features (age and gender).
- The **MIMIC-IV dataset**^12^ is sourced from the EHRs of the Beth Israel Deaconess Medical Center. We utilize version 3.1 of its structured EHR data^15^ and version 2.2 of its clinical notes (discharge summaries)^16^.
- The **MIMIC-III dataset**^13^ is an earlier version from the same medical center. We use version 1.4^13^ to extract admission notes for our prospective mortality prediction task.

Our preprocessing methodology adheres to established benchmark pipelines^17,18^. For MIMIC-III and MIMIC-IV, each unique hospital admission (identified by subject and admission IDs) was treated as a distinct sample. To address the critical issue of potential data contamination, where models (particularly proprietary LLMs with opaque training corpora) might have been inadvertently trained on benchmark data, we provide a detailed timeline analysis in Supplementary Information.

For the structured EHR data, to mitigate issues arising from missing values, we first consolidate all data segments from the same admission on a daily basis. In MIMIC-IV dataset, we take the last recorded value for each of the 17 physiologic variables within a 24-h window. For patients whose hospital stays exceed seven days, we retain records exclusively from the final seven days, while earlier records are aggregated into a single initial time step (also using the last recorded value for each feature). For conventional ML/DL models, any remaining missing values are addressed using the Last Observation Carried Forward (LOCF) imputation strategy^19^. For LLMs, missing values are explicitly passed as “NaN”. Structured data were standardized using z-score normalization, and extreme outliers (absolute z-score >10,000) were removed.

Regarding the clinical notes, we used discharge summaries from MIMIC-IV and admission notes from MIMIC-III. To create the admission notes cohort from MIMIC-III, we extracted all “Physician”, “Nursing”, and “Nursing/other” notes recorded within the first 24 h of admission and concatenated them chronologically. The preprocessing of all notes follows the Clinical-Longformer approach^20^, which involves several key steps: removal of all de-identification placeholders, replacement of non-alphanumeric characters with spaces, conversion of all text to lowercase, and stripping of extraneous white spaces. Critically, to prevent label leakage, we programmatically removed any explicit mentions of outcomes (e.g., “expired”, “deceased”, “readmitted”) from the notes.

Furthermore, consistent with benchmark practices, we employ a stratified shuffling strategy with random selection to partition the data into training, validation, and test sets. Crucially, the test set is held constant across all evaluated models to ensure a fair and robust comparison of performance. Detailed statistics for all data modalities are presented in Table 6. The complete preprocessing code is publicly available at https://github.com/PKU-AICare/mimic_preprocessor/.

**Table 6:** Statistics of the TJH dataset, MIMIC-III Note (clinical notes), MIMIC-IV EHR (structured EHR data) and MIMIC-IV Note (clinical notes) datasets

Dataset	TJH			MIMIC-III Note			MIMIC-IV EHR					MIMIC-IV Note				
	Total	Alive	Dead	Total	Alive	Dead	Total	Alive	Dead	Re.	No Re.	Total	Alive	Dead	Re.	No Re.
*Test Set Statistics*																
# Patients	200	109	91	200	180	20	200	183	17	53	147	200	183	17	53	147
# Total visits	967	601	366	200	180	20	801	717	84	274	527	200	183	17	53	147
# Avg. visits	4.8	5.5	4.0	1.0	1.0	1.0	4.0	3.9	4.9	5.2	3.6	1.0	1.0	1.0	1.0	1.0
Avg. LOS	7.1	7.8	5.8	-	-	-	-	-	-	-	-	-	-	-	-	-
*Training Set Statistics*																
# Patients	140	75	65	8750	7887	863	8750	8028	722	2112	6638	8750	8028	722	2112	6638
# Total visits	641	395	246	8750	7887	863	33423	30117	3306	10,448	22,975	8750	8028	722	2112	6638
# Avg. visits	4.6	5.3	3.8	1.0	1.0	1.0	3.8	3.8	4.6	4.9	3.5	1.0	1.0	1.0	1.0	1.0
Avg. LOS	8.6	9.0	8.0				-	-	-	-	-	-	-	-	-	-
*Validation Set Statistics*																
# Patients	21	11	10	1250	1127	123	1250	1147	103	305	945	1250	1147	103	305	945
# Total visits	96	54	42	1250	1127	123	4685	4176	509	1522	3163	1250	1147	103	305	945
# Avg. visits	4.6	4.9	4.2	1.0	1.0	1.0	3.7	3.6	4.9	5.0	3.3	1.0	1.0	1.0	1.0	1.0
Avg. LOS	6.5	8.1	4.4	-	-	-	-	-	-	-	-	-	-	-	-	-

"Re.” stands for Readmission, indicating patients who are readmitted to the ICU within 30 days of discharge, while “No Re.” represents patients who are not readmitted. “LOS” denotes “length-of-stay”.

### Benchmarking task formulation

Following previous clinical prediction benchmarking studies^1,17^, we chose three widely recognized clinical tasks to assess model performance. To ensure reproducibility and clarity, we provide precise specifications for each task, including the observation period, prediction timepoint, and whether the task is prospective (predicting a future event) or retrospective (classifying an event that has already occurred based on a complete record). A summary is provided in Table 7, with detailed definitions below.

- **In-hospital mortality prediction:** A binary classification task to predict whether a patient will die during their hospital stay. The label is 1 if a patient’s recorded time of death is between their admission and discharge times; otherwise, it is 0. This task is evaluated under three distinct settings:
  - *Retrospective (Structured EHR):* Using structured EHR data from the entire hospital stay on both MIMIC-IV and TJH datasets, with the prediction made at the time of discharge.
  - *Retrospective (Discharge Notes):* Using the complete discharge summary from MIMIC-IV, which summarizes the entire hospitalization, with the prediction made at the time of discharge. We acknowledge that discharge summaries are generated post-outcome and contain inherent temporal leakage. Thus, this setting evaluates the model’s ability to classify outcomes based on linguistic cues and medical content (document classification) rather than predicting future events.
  - *Prospective (Admission Notes):* Using clinical notes recorded within the first 24 hours of admission from the MIMIC-III dataset to predict future mortality, making this a true prospective task.
- **30-day readmission prediction:** A prospective binary classification task to predict whether a patient will be readmitted within 30 days of discharge. The label is 1 if a subsequent admission occurs within this window. Following established benchmarks, if a patient dies within 30 days post-discharge, this is also counted as a positive readmission event. The prediction is made at the time of discharge using either structured EHR data or discharge notes from MIMIC-IV. This task was evaluated exclusively on the MIMIC-IV dataset, as the TJH dataset was specifically curated for an in-hospital mortality prediction study and does not contain the necessary post-discharge follow-up data required for a readmission analysis^14^.
- **Length-of-stay (LOS) prediction:** A prospective regression task to predict the remaining duration of a patient’s hospital stay in days. The prediction is made dynamically at each patient visit using cumulative structured EHR data from the TJH dataset, allowing for early intervention.

**Table 7:** Summary of task specifications, datasets, and timepoints

Task	Dataset	Data Modality	Observation Window	Prediction Timepoint	Prediction Target
In-hospital Mortality	MIMIC-IV / TJH	Structured EHR	In-hospital since admission	At discharge (Retrospective)	Mortality during the hospital stay
	MIMIC-IV	Discharge Notes	At discharge	At discharge (Retrospective)	Document classification of mortality status
	MIMIC-III	Admission Notes	First 24 h of admission	Within 24 h of admission (Prospective)	Mortality during the hospital stay
30-day Readmission	MIMIC-IV	Structured EHR	In-hospital since admission	At discharge (Prospective)	Readmission within 30 days post-discharge
	MIMIC-IV	Discharge Notes	At discharge	At discharge (Prospective)	Readmission within 30 days post-discharge
Length-of-Stay	TJH	Structured EHR	In-hospital since admission	At each patient visit (Prospective)	Remaining duration of hospital stay (in days)

This table provides a clear and rigorous definition for each prediction task, specifying the data used, the observation window, the precise timepoint at which a prediction is made, and the prediction target.

### Benchmarking model selection

We examined a diverse array of state-of-the-art models to ensure generalizable and comprehensive conclusions, spanning machine learning and deep learning-based clinical prediction models, BERT-style language models, and GPT-style LLMs, including base LLMs and advanced reasoning LLMs:

- *Conventional ML/DL models for clinical predictions* included four conventional machine learning models (CatBoost^21^, Decision tree, Random Forest^22^, XGBoost^23^) and three deep learning models (GRU^24^, LSTM^25^, RNN^26^), as well as advanced predictive models designed for longitudinal EHR data (AdaCare^27^, ConCare^2^, GRASP^28^, AICare^29^).
- *BERT-style models* included BERT models pretrained on general texts (BERT^30^) and the biomedical corpus (ClinicalBERT^31^, BioBERT^32^, GatorTron^33^, Clinical-Longformer^20^). We note that the specialized BERT-style models (ClinicalBERT, GatorTron, Clinical-Longformer) included MIMIC-III clinical notes in their pretraining corpora. Given that the precise patient or admission identifiers included in these pretraining corpora are not publicly documented in the model releases, strict data decontamination was infeasible. Consequently, these models represent an optimistic upper bound for BERT-style architectures, serving as a strong in-domain reference point.
- Large language models included several general purpose LLMs (GPT-2^34^, GPT-4o^35^, Gemma-3^36^, Qwen2.5^37^, and DeepSeek-V3.1^38,39^), along with medically finetuned variants (BioGPT^40^, Meditron^41^, OpenBioLLM^42^, and BioMistral^43^). We also include advanced reasoning LLMs, such as HuatuoGPT-o1-7B^44^, DeepSeek-V3.1-Think^38,39^, DeepSeek-R1 (version 250528)^45^, GPT-5 (High)^46^, DeepSeek-R1-Distill-Qwen^45^, and GPT o3-mini-high^47^).

We summarized the details of all baseline models in Table 8. Throughout the experiments, we strictly adhered to the data use agreement. The performance of OpenAI models on all datasets was processed using the secure Azure OpenAI API, with human review of the data waived. Additionally, all other models, including ML, DL, and other LLMs, were deployed locally. The code for this benchmarking work, including all preprocessing scripts and prompt templates, can be accessed online (https://github.com/yhzhu99/ehr-llm-benchmark).

**Table 8:**
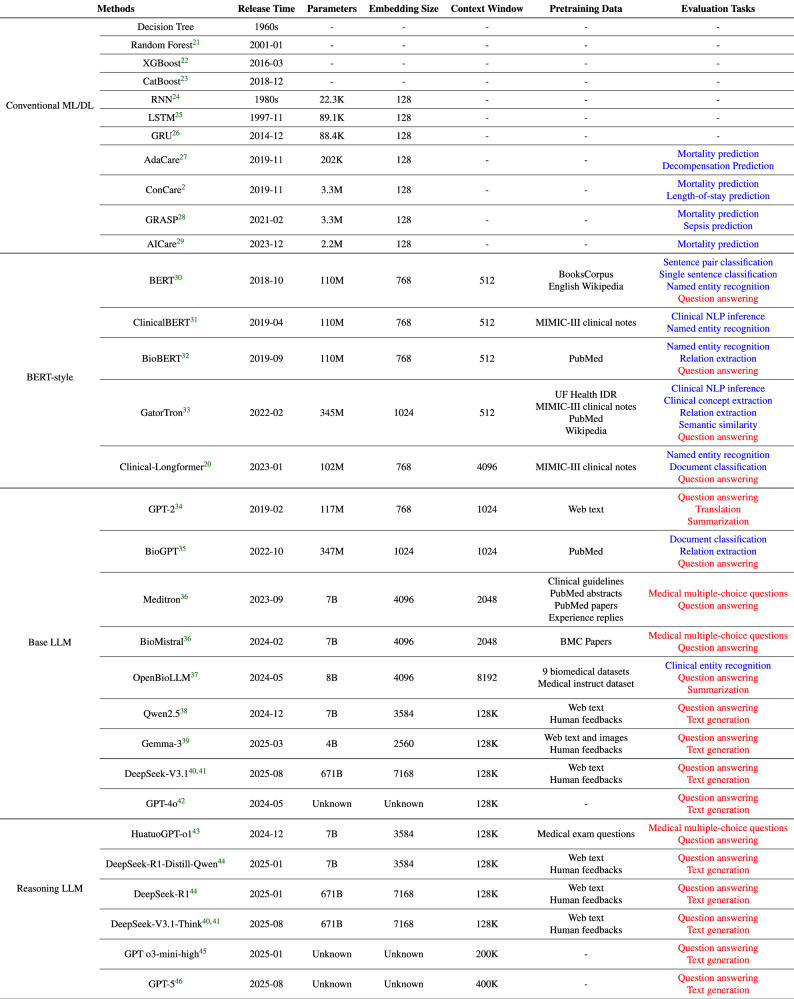
Baseline models compared in this benchmark, including conventional ML/DL models, BERT-style models, Base LLMs, and Reasoning LLMs

Conventional ML/DL models focus on clinical predictive tasks utilizing structured EHR data. BERT-style models primarily process unstructured clinical notes. LLMs, being general-purpose, are suitable for evaluation across both structured EHR data and unstructured clinical notes. Non-generative tasks are highlighted in **Blue**, while generative tasks are marked in **Red**. In the case of GPT-style models, tasks like document classification, though inherently classification tasks, are conducted in a generative manner through the use of prompts.

Given that the decoder-only architecture of LLMs, trained primarily on unstructured natural language texts, encounters challenges with the structured, sparse, and longitudinal nature of EHR data^48,49^, we utilized a prompting strategy to better deliver structured EHR data to LLMs. The prompting strategy employed a feature-wise list-style format for inputting EHR data and provided LLMs with feature units and reference ranges. We manually looked up the unit and reference ranges for each clinical feature in the medical guidelines. We also explored the in-context learning strategy in the prompt by providing example cases. The prompt templates for the prediction tasks with EHR data are shown in Supplementary Information.

### Multimodal integration experiments

Recognizing that a holistic patient view often requires synthesizing information from multiple sources, we designed a set of experiments to evaluate model performance on multimodal data. We combined structured EHR time-series data with unstructured discharge summaries from the MIMIC-IV dataset for both the in-hospital mortality and 30-day readmission prediction tasks. We assessed two primary integration strategies: a prompt-based approach for zero-shot LLMs and a feature-fusion approach for finetuned models.

For LLMs, we developed a unified prompt that presents both structured EHR data and the clinical note in a single context, testing the models’ inherent ability to synthesize information from disparate data types. The structured data was formatted as a time-series list, followed by the full text of the clinical note. The specific templates are provided in the Supplementary Information.

For finetuned models, we adopted a feature-fusion strategy. This involved first generating high-quality vector embeddings for each modality using the best-performing unimodal encoders identified in our main experiments. The resulting embeddings were then combined using four standard fusion techniques:

- **Addition**: Element-wise summation of the embedding vectors.
- **Concatenation**: Appending the vectors to form a single, larger representation.
- **Self-Attention**: Applying a multi-head self-attention mechanism to each embedding independently before concatenation.
- **Cross-Attention**: Employing a bidirectional multi-head attention mechanism to allow the embeddings from each modality to mutually inform one another before concatenation.

A final classification layer was then trained on these fused representations.

### Evaluation settings

When evaluating the performance on unstructured clinical notes data, three settings were considered for evaluation: freeze, finetune, and prompt. In the freeze setting, we use the pretrained model to generate text embeddings and train a classifier on these embeddings to assess the out-of-the-box performance of the language models. For feature extraction, we utilized the embedding of the first token ([CLS]) from the final layer of BERT-style models and the embedding of the last token from the final layer for GPT-style models. The finetune setting involves further finetuning of the language model parameters together with the classifier training process. The prompt setting instructs the LLMs to directly generate prediction results from clinical notes. We use the instruction-tuned version of LLMs to make sure the task can be properly understood. The LLMs were also encouraged to output their thinking process before the final prediction. This was a deliberate methodological choice to simulate a realistic clinical decision-support scenario, where a standalone probability score is insufficient to build trust or guide practice^50,51^. While prompting for only a numerical output can simplify automated parsing^52^, it disconnects the model’s output from the essential clinical workflow of evidence-based reasoning. The templates for the prediction tasks with clinical notes data are shown in Supplementary Information.

For proprietary models (e.g., GPT-5) or large-scale models (e.g., DeepSeek-R1-671B), we only evaluated them under the prompt setting. For structured EHR data, the length of the prompt can reach several thousand tokens. Due to cost issues, we only evaluate them under the prompt setting to show their out-of-the-box clinical prediction ability. For a fair comparison against the LLMs without further domain-specific training (zero-shot performance), the ML/DL models were evaluated after minimal training (few-shot setting). Following the common medical few-shot learning settings^53^, the models were trained using only 10 samples (5 positive and 5 negative cases). We also provided the performance trained on the full dataset for reference. Additionally, we conducted multimodal integration analyses using both MIMIC-IV’s structured EHR and clinical notes data, exploring fusion strategies to assess combined predictive power.

The classification tasks (mortality and readmission prediction) were evaluated using the Area Under the ROC Curve (AUROC) and the Area Under the Precision-Recall Curve (AUPRC). The regression task (LOS prediction) was evaluated using mean absolute error (MAE), mean squared error (MSE), and root mean squared error (RMSE).

To ensure a robust interpretation of our results, we report all performance metrics with 95% confidence intervals (CIs) computed via a bootstrapping methodology. For each experiment, we drew 100 bootstrap samples with replacement from the test set. The performance metric was calculated for each sample, and from the resulting distribution of 100 values, we report the mean and standard deviation. The 95% CI is defined by the 2.5th and 97.5th percentiles of this distribution. Results in all tables are presented in the format: ${{\rm{mean}}}_{[2.5 \% ,97.5 \% ]}^{\,{\rm{std}}}$.

In addition to these performance metrics, we conducted a comprehensive fairness analysis to assess potential model biases across key demographic subgroups. The detailed methodology and results of this ethical evaluation are presented in Supplementary Information. The detailed experiment settings can be found in Supplementary Information.

A critical aspect of our evaluation was the systematic handling of instances where an LLM failed to follow prompt instructions. We established a protocol to ensure fair and reproducible comparisons. Minor formatting errors (e.g., a missing comma in a JSON output) were manually corrected to extract the valid prediction. For more significant failures where a model did not provide a prediction (a “failure to predict”), we did not discard the sample. Instead, we imputed a default, non-informative value to penalize the model appropriately in the performance metrics. For binary classification tasks, we imputed a probability of 0.5 (equivalent to random chance), and for the regression task, we imputed the median value from the training set. This protocol ensures that all models are evaluated on the identical test set, with less reliable models being impacted accordingly.

To validate our prompting strategy and assess the clinical utility of the LLM-generated explanations, we conducted a formal human evaluation study. This qualitative analysis was designed to move beyond predictive accuracy and measure the quality, safety, and usefulness of the models’ reasoning, which is critical for their adoption in high-stakes clinical environments^54,55^.

We recruited a panel of five clinicians with expertise in internal and critical care medicine. We created a diverse case portfolio by randomly sampling 25 cases from the test set for each of the two primary prediction tasks (in-hospital mortality and 30-day readmission) across both data modalities (MIMIC-IV structured EHR and MIMIC-IV discharge notes). This resulted in a total pool of 100 unique patient cases. For each case, we selected the output from the best-performing LLM identified in our main experiments for that specific task setting.

**Fig. 3:**
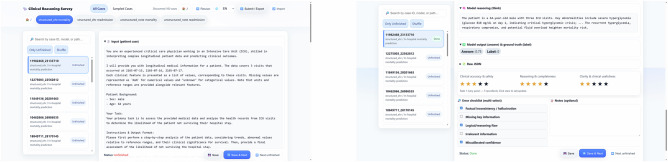
Web-based interface for the human evaluation study. Expert evaluators were presented with the patient’s clinical data (input), the LLM’s generated reasoning and prediction, and the ground truth label. They used the interface to provide scores on a 1–5 Likert scale for three quality dimensions and to select predefined error types from a checklist.

Each expert evaluated a random subset of 20 cases (5 from each setting). For each case, the experts were presented with the complete input provided to the LLM (either the structured EHR data or the clinical note), the model’s full, unaltered output (both the reasoning and the final prediction score), and the ground truth label. The evaluation was conducted using a custom-built, secure web-based annotation system (Fig. 3), where experts were presented with the source data, the LLM’s full output, and the ground truth label. A public-facing demo of the system is available online (https://yhzhu99.github.io/ehr-llm-benchmark/human-eval.html). The framework included two components:

- **Quantitative Scoring:** Experts rated each explanation on a 1-5 Likert scale across three dimensions:
  1. **Clinical Accuracy and Safety**. How clinically sound and factually consistent is the reasoning with the provided source data? Does it contain factually incorrect statements or clinically unsafe assertions? (1 = Very inaccurate/Unsafe, 5 = Highly accurate/Safe).
  2. **Reasoning and Completeness**. Does the model identify and logically connect the most important risk factors to its prediction? Does it omit critical information that a clinician would consider essential? (1 = Illogical/Incomplete, 5 = Logical/Comprehensive).
  3. **Clarity and Clinical Utility**. Is the explanation clear, concise, and presented in a way that would genuinely aid a clinician’s decision-making process? (1 = Unclear/Useless, 5 = Very clear/Useful).
- **Qualitative Error Checklist:** Experts identified the presence of specific, predefined error types from a comprehensive taxonomy (Fig. 2) developed through an iterative open-coding process by two clinicians on a pilot set of cases. The primary error categories included:
  1. **Factual Inconsistency/Hallucination**. The reasoning includes information that is absent from or directly contradicts the provided patient record (e.g., citing a non-existent comorbidity or an incorrect lab value). This is a form of hallucination.
  2. **Omission of Key Information**. The reasoning fails to mention critical patient data that is highly relevant to the prediction (e.g., ignoring a recent lab result indicating acute kidney injury when assessing mortality risk).
  3. **Flawed Logic or Reasoning**. The facts cited from the patient record are correct, but the clinical conclusion drawn from them is unsound, illogical, or represents a misinterpretation of their significance (e.g., incorrectly linking a stable chronic condition to an acute risk).
  4. **Inclusion of Irrelevant Information**. The reasoning focuses on non-contributory or distracting details from the patient record, which can obscure the key risk factors and weaken the clinical utility of the explanation.
  5. **Inappropriate Confidence**. The model expresses a level of certainty that is not clinically warranted. This can manifest as overconfidence in a speculative conclusion or, conversely, as undue hesitancy regarding a clear and significant risk factor.
  To ensure consistency and reduce subjectivity, we provided clinicians with an explicit, anchored rubric and an evaluation instruction script, detailed in the Supplementary Information.

### Computational infrastructure and model settings

The computational infrastructure and specific model settings employed for our experiments are detailed below to ensure full reproducibility. Our LLM generation experiments were conducted from April 10, 2025, to April 24, 2025, and from September 6, 2025, to September 21, 2025. For prediction tasks involving structured EHR data, both the training of machine learning/deep learning models and the LLM generation experiments were performed on a server equipped with 128GB of RAM and a single NVIDIA RTX 3090 GPU (CUDA 12.5). For prediction tasks utilizing clinical notes: (1) Experiments with LLMs in the freeze setting (training only the MLP classifier head) and the finetuning of BioGPT were conducted on a Mac Studio M2 Ultra with 192GB of RAM for MIMIC-IV dataset, and on a Mac Studio M3 Ultra with 512GB of RAM for MIMIC-III dataset. (2) Finetuning of the other LLMs was carried out on a system featuring an NVIDIA A100 GPU (80GB VRAM) and 64GB of RAM for MIMIC-IV dataset, and on the same Mac Studio M3 Ultra with 512GB of RAM for MIMIC-III dataset. (3) LLM inference experiments using prompting methodologies were executed on the same NVIDIA RTX 3090 server used for the structured EHR data tasks for MIMIC-IV dataset, and on a MacBook M3 Air with 24GB of RAM for MIMIC-III dataset. The primary software stack comprised Python 3.12, PyTorch 2.6.0, PyTorch Lightning 2.5.1, and Transformers 4.50.0.

Regarding model training and hyperparameters, across all experiments, the AdamW optimizer^56^ was employed. For conventional EHR prediction models, training proceeded for a maximum of 50 epochs on the designated training set. To mitigate overfitting, an early stopping strategy was implemented with a patience of 5 epochs, monitored by AUROC for classification tasks and MAE for the regression task. The learning rate was selected via grid search from the set {1 × 10^−2^, 1 × 10^−3^, 1 × 10^−4^}. These models utilized a hidden dimension of 128 and a batch size of 256. For language models (LMs), the following training configurations were applied: (1) *Freeze Setting*: The MLP classifier head appended to each LM was trained for a maximum of 50 epochs with an early stopping patience of 10 epochs. The learning rate was set to 1 × 10^−4^, and the batch size is 64. (2) *Finetuning BERT-style LMs*: These models were finetuned for a maximum of 10 epochs with an early stopping patience of 3 epochs. The learning rate was 1 × 10^−5^, and the batch size is 16. (3) *Finetuning GPT-style LLMs*: These models were finetuned for a maximum of 5 epochs with an early stopping patience of 1 epoch. The learning rate was 2 × 10^−4^, and the batch size is 8. For feature extraction from LMs, we utilized the embedding of the first token ([CLS]) from the final layer of BERT-style models. For GPT-style models, the embedding of the last token from their final layer was used. In experiments involving clinical notes (both frozen backbone and finetuning settings), the maximum input sequence length was set to 512 tokens for all LMs. For LLM generation experiments, this was expanded to 8192 tokens to leverage the models’ capacity for longer contexts.

For language model access and deployment, we utilized OpenAI’s APIs, including the GPT-5 (gpt-5-chat-latest with reasoning effort of high), the GPT-4o (chatgpt-4o-latest) and GPT o3-mini-high (o3-mini-high) models. All other LMs evaluated in this study were deployed locally. LLM generation experiments using these local models for both structured EHR data and unstructured clinical notes were facilitated by LMStudio. For the freeze and finetuning experiments, LMs were loaded using the Hugging Face Transformers library without any additional quantization, thereby preserving their original precision and computational capabilities. The detailed model settings and quantized versions fetched from HuggingFace are listed in Supplementary Information.

## Data Availability

To ensure the fairness and reproducibility of the comparison, this research did not involve the collection of new patient EHR data. The TJH EHR dataset utilized in this study is publicly available on GitHub (https://github.com/HAIRLAB/Pre_Surv_COVID_19). The MIMIC-IV datasets are open to researchers and can be accessed on request, including structured EHR data^15^ (https://physionet.org/content/mimiciv/3.1/) and clinical notes data^16^ (https://physionet.org/content/mimic-iv-note/2.2/). We used these datasets under their respective licenses. Throughout the experiments, we strictly adhered to the data use agreement, reaffirming our commitment to responsible data handling and usage. The performance of OpenAI models on all datasets was processed using the secure Azure OpenAI API, with human review of the data waived. Additionally, all other models, including ML, DL, and other LLMs, were deployed locally.

## Code availability

The code for this benchmarking work can be accessed online (https://github.com/yhzhu99/ehr-llm-benchmark). We also provide the up-to-date benchmark results online (https://yhzhu99.github.io/ehr-llm-benchmark/).
